# *Pneumocystis* pneumonia risk among viral acute respiratory distress syndrome related or not to COVID 19

**DOI:** 10.1186/s13054-021-03767-3

**Published:** 2021-09-26

**Authors:** Keyvan Razazi, Romain Arrestier, Anne Fleur Haudebourg, Francoise Botterel, Armand Mekontso Dessap, and the COVID PCP group, Slim Fourati, Brice Benelli, Frédérique Boquel, Nicolas de Prost, Guillaume Carteaux, Jeanne Tran Van Nhieu, Frederic Schlemmer

**Affiliations:** 1 Service de Médecine Intensive Réanimation, AP-HP (Assistance Publique-Hôpitaux de Paris), Hôpitaux Universitaires Henri Mondor, DMU Médecine, 94010 Créteil, France; 2 Faculté de Santé de Créteil, IMRB, GRC CARMAS, UPEC (Université Paris Est Créteil), 94010 Créteil, France; 3 Département de Virologie, Bactériologie, Parasitologie-Mycologie, AP-HP (Assistance Publique-Hôpitaux de Paris), Hôpitaux Universitaires Henri Mondor, 94010 Créteil, France; 4 EA 7380 Dynamic, UPEC, Ecole Nationale Vétérinaire d’Alfort, USC Anses, Créteil, France; 5 INSERM, Unité U955, France Université Paris Est, 94010 Créteil, France; 6 Service de Medecine Intensive Réanimation, CHU Henri Mondor, 51, Av de Lattre de Tassigny, 94000 Créteil Cedex, France

Lymphopenia, corticosteroids and immunomodulatory therapeutics frequently used in COVID-19 patients with acute respiratory distress syndrome (C-ARDS) may be contributing factors to opportunistic infection such as *Pneumocystis jirovecii* pneumonia (PCP).

We conducted a retrospective study to compare the prevalence of PCP between patients with C-ARDS and those with non-SARS-CoV-2 viral ARDS (NC-ARDS).

Methods and some data from this cohort have been previously published [1]. There was no systematic protocol to search for PCP but in case of suspicion of PCP (respiratory symptoms with any consistent radiographic features), several analyses were performed on respiratory samples, such as broncho-alveolar lavage (BAL), blind protected sample, or sputum. It included direct examination (using May-Grünwald Giemsa (MGG), or immunofluorescence staining), detection of *Pneumocystis jirovecii* DNA by real-time polymerase chain reaction (qPCR) [2], and serum (1–3)-β-D-glucan. During the COVID-19 outbreak, immunofluorescence staining was not performed. PCP was defined as per the revised EORTC/MSGERC definition [3] as follows: proven in case of suspicion with positive direct examination; possible in case of suspicion with positive qPCR and positive BDG in ≥ 2 consecutive serum samples provided other etiologies have been excluded. SARS-CoV-2 and other viruses were not considered a priori as host factors. Patients with positive qPCR but lacking the other criteria for possible PCP were classified as colonized.

The primary endpoint was the difference in prevalence of PCP between C-ARDS and NC-ARDS patients.

No statistical sample size calculation was performed a priori, and sample size was equal to the number of patients treated during the study period. All patients were included only once.

Between October 1, 2009, and April 29, 2020, ninety patients had C-ARDS (positive RT PCR test for SARS-CoV-2), while 82 patients had viral NC-ARDS. Our study comprises 120 patients with fungal analyses on respiratory samples obtained from 81 C-ARDS and 39 NC-ARDS patients. NC-ARDS patients had more comorbidities were more often immunocompromised, and had lower lymphocyte counts than C-ARDS patients (Table 1). C-ARDS patient received less steroid than NC-ARDS patients because they were included before randomized trials demonstrating decreased mortality with dexamethasone.

**Table 1:** Characteristics of patients with *Pneumocystis jirovecii* research according to C-ARDS and NC-ARDS patients

	NC-ARDS (n = 39)	C-ARDS (n = 81)	p-value
Age, median [IQR]	61.8 [56.1–69.3]	58 [52–69.5]	0.32
Male gender	28 (72%)	65 (80%)	0.30
Medical history			
Mc Cabe			< 0.0001
No underlying condition	13 (33%)	70 (86%)	
Ultimately fatal	16 (41%)	10 (12%)	
Rapidly fatal disease	10 (26%)	1 (1%)	
Charlson comorbidity index	3 [2–4]	1 [0–2]	< 0.0001
Diabetes mellitus	11 (28%)	38 (47%)	0.051
Congestive heart failure (NYHA 3–4)	3 (8%)	6 (7%)	> 0.99
Supraventricular arrhythmia	5 (13%)	8 (10%)	0.76
Hypertension	16 (41%)	52 (64%)	0.016
COPD	2 (5%)	8 (10%)	0.50
Chronic renal failure	8 (21%)	13 (16%)	0.55
Dialysis	3 (8%)	2 (3%)	0.33
Stroke	1 (3%)	3 (4%)	> 0.99
Liver cirrhosis (Child C)	1 (3%)	0 (0%)	0.33
Current smoking	8 (21%)	21 (26%)	0.52
Immunosuppression conditions			
Overall	32 (82%)	13 (16%)	< 0.0001
Solid cancer	2 (5%)	4 (5%)	> 0.99
Blood cancer	15 (38%)	0 (0%)	< 0.0001
Organ transplant	9 (23%)	5 (6%)	0.013
HIV infection	3 (8%)	3 (4%)	0.39
Sickle cell disease	1 (3%)	3 (4%)	> 0.99
Others	4 (10%)	1 (1%)	0.038
Clinical characteristics upon ICU admission			
IGS2	51 [37–68]	35 [27–43]	< 0.0001
Baseline SOFA, median [IQR]	9 [6–12]	7 [4–8]	< 0.0001
ARDS classification (Berlin definition)			0.046
Mild	12 (31%)	10 (12%)	
Moderate	18 (46%)	44 (54%)	
Severe	9 (23%)	27 (33%)	
Norepinephrine, n (%)	20 (51.3%)	35 (43.2%)	0.41
Serum creatinine (µmol/L)	147 [83–226]	82 [66–124]	0.001
White blood cell count (× 10^9^/L)	5.4 [3–14.8]	8.1 [5.5–11.9]	0.44
Lymphocyte count (× 10^9^/L)	0.4 [0.2–0.9]	0.8 [0.5–1.2]	0.01
Documented bacterial co-infections	18 (46%)	13 (16%)	< 0.0001
Treatments during the first 24 h			
Antibiotics	39 (100%)	81 (100%)	> 0.99
Antiviral treatment	26 (67%)	65 (80%)	0.10
Corticosteroids (any dose)	21/38 (55%)	10/79 (13%)*	< 0.0001
Corticosteroids (low dose)	20/38 (53%)	8/79 (10%)*	< 0.0001
Corticosteroids (high dose) #	1/38 (3%)	2/79 (3%)*	> 0.99
ARDS treatment during ICU stay			
Corticosteroids (any dose)	24 (63%)	32 (41%)*	0.02
Corticosteroids (low dose)	22 (58%)	22 (28%)*	0.002
Corticosteroids (high dose) #	2 (5%)	10 (13%)*	0.22
Prone position	20 (51%)	71 (88%)	< 0.0001
Neuromuscular blockade	25 (64%)	74 (91%)	< 0.0001
Inhaled nitric oxide	6 (15%)	28 (35%)	0.03
Extra-corporeal membrane oxygenation	5 (13%)	20 (25%)	0.13
Organ support and outcome during ICU stay			
Renal replacement therapy during ICU stay	19 (49%)	29 (36%)	0.18
Norepinephrine, n (%)	32 (82%)	61 (75%)	0.41
ICU length of stay among survivors, days	17 [10–28]	30 [22–46]	0.09
Death at day 28	15 (39%)	30 (37%)	0.88
Death in the ICU	17 (44%)	32 (40%)	0.67
*Pneumocystis jirovecii* samples and analysis			
Total samples, mean (range)	1.5 (1–4)	3.8 (1–15)	< 0.001
Sputum examination, mean (range)	0.08 (0–1)	0	0.01
Broncho-alveolar lavage, mean (range)	1.5 (0–4)	0.19 (0–2)	< 0.001
Blind protected sample, mean (range)	0	3.6 (1–15)	< 0.001
Direct examination (IF or MGG)	1.5 (0–4)	0.19 (0–2)	< 0.001
qPCR	1.5 (1–4)	3.8 (1–15)	< 0.001
Serum (1–3)-BDG	0.5 (0–4)	4 (1–10)	< 0.001

COPD = chronic obstructive pulmonary disease, HIV = human immunodeficiency virus, SAPS II = Simplified Acute Physiology Score II, SOFA = sequential organ failure assessment, ICU = intensive care unit; *two missing values because two patients received dexamethasone or placebo in a randomized controlled trial; #denotes more than 1 mg/kg of prednisone or equivalent

*Pneumocystis* analyses were performed on a mean of 3.1 respiratory sample per patient (range 1–15). Direct examination was performed in a total of 72 samples, with two positive cases. qPCR was performed in a total of 368 samples (294 blind protected samples, 72 BAL, and three sputum). All qPCR were negative in C-ARDS patients, while five (13%) NC-ARDS patients had at least one positive PCR, with a median cycle threshold of 36.6 [30–38.3].

Two NC-ARDS patients fulfilled proven PCP diagnostic criteria, with a positive direct examination, a single ß-D-glucan > 80 pg/mL (Table 2), and received treatment for PCP.

**Table 2:** Characteristics of patients with a positive *Pneumocystis jirovecii* qPCR

Patient, age, sex	Underlying disease	Date of PCP diagnosis	Viral association	Respiratory sample	Direct examination (IFI or MGG)	Pneumocystis qPCR	BDG (pg/ml)*	Time between ICU admission and positive sample (day)	Treatment
P1, 58y, M	Diabetus mellitus Congestive heart failure	14/01/2014	Coronavirus, Rhinovirus	BAL	Negative	36.7	NA	1	No
P2, 73y, M	Renal transplantation Diabetus mellitus Congestive heart failure	29/08/2015	Coronavirus	BAL	Positive	32	106	0	Yes (sulfamethoxazole)
P3, 52y, M	Myasthenia (steroid, azathioprine)	04/07/2012	Respiratory syncytial virus	BAL	Negative	39.8	NA	1	No
P4, 32y, F	Acute lymphoblastic leukemia (methotrexate and aracytabine)	08/01/2019	Metapneumovirus	BAL	Positive	27.9	188	0	Yes (sulfamethoxazole)
P5, 67y, M	Cirrhosis, rheumatoid polyarthritis (steroid)	22/04/2019	Coronavirus NL63	BAL	Negative	36.6	NA	1	No

M = male, F = female, P = patient; BDG = (1–3)-β-D-glucan,*BDglucan not performed in the lab before 2013. BDglucan was performed using the Fungitell kit™ (Cape Cod Inc, USA) with a positivity threshold of 80 pg/mL; qPCR of *P.jirovecii* was performed using a region of the mitochondrial large subunit rRNA gene (LSU) after DNA extraction with a Qiasymphony kit (Qiagen, Courtaboeuf, France)

Three other NC-ARDS patients were classified as colonized, while no patient fulfilled possible PCP diagnostic criteria. Time between ICU admission and positive sample for PCP (Table 2) was short (< 2 days) like in other invasive fungal infections (i.e. invasive pulmonary aspergillosis) in severe influenza infection or ARDS.

In this study of patients with viral ARDS, we found a low risk for possible or proven PCP. Our findings are in accordance with two smaller studies in France [4, 5] retrieving a low risk of *Pneumocystis* colonisation in COVID-19 patients. In our cohort, qPCR was positive in 13% of NC-ARDS. This result is in accordance with a previous study showing 7% of positive qPCR in ICU-admitted influenza patients [6].

The strengths of our study are the analysis of a large ARDS cohort with fungal analyses. Our study also has limitations: monocentric design, NC-ARDS patients more frequently immunocompromised, and a long cohort period.

## Data Availability

The datasets supporting the conclusions are included within the article.

## Abbreviations

C-ARDS: Coronavirus disease 19 related acute respiratory distress syndrome
NC-ARDS: Non-coronavirus disease 19 viral ARDS
PCP: *Pneumocystis jirovecii* Pneumonia
